# Impact of hematological inflammatory markers on clinical outcome in patients with salivary duct carcinoma: a multi-institutional study in Japan

**DOI:** 10.18632/oncotarget.13565

**Published:** 2016-11-24

**Authors:** Daisuke Kawakita, Yuichiro Tada, Yorihisa Imanishi, Shintaro Beppu, Kiyoaki Tsukahara, Satoshi Kano, Hiroyuki Ozawa, Kenji Okami, Yuichiro Sato, Akira Shimizu, Yukiko Sato, Chihiro Fushimi, Soichiro Takase, Takuro Okada, Hiroki Sato, Kuninori Otsuka, Yoshihiro Watanabe, Akihiro Sakai, Koji Ebisumoto, Takafumi Togashi, Yushi Ueki, Hisayuki Ota, Tomotaka Shimura, Toyoyuki Hanazawa, Shingo Murakami, Toshitaka Nagao

**Affiliations:** ^1^ Department of Otorhinolaryngology, Head and Neck Surgery, Nagoya City University Graduate School of Medical Sciences, Nagoya, Japan; ^2^ Department of Head and Neck Oncology and Surgery, International University of Health and Welfare Mita Hospital, Tokyo, Japan; ^3^ Department of Otorhinolaryngology-Head and Neck Surgery, Keio University School of Medicine, Tokyo, Japan; ^4^ Department of Otolaryngology, Tokyo Medical University School of Medicine, Tokyo, Japan; ^5^ Department of Otorhinolaryngology-Head and Neck Surgery, Hokkaido University Graduate School of Medicine, Sapporo, Japan; ^6^ Department of Otolaryngology-Head and Neck Surgery, Tokai University School of Medicine, Isehara, Japan; ^7^ Department of Head and Neck Surgery, Niigata Cancer Center Hospital, Niigata, Japan; ^8^ Department of Pathology, Cancer Institute Hospital, Japanese Foundation for Cancer Research, Tokyo, Japan; ^9^ Department of Anatomic Pathology, Tokyo Medical University School of Medicine, Tokyo, Japan; ^10^ Department of Otolaryngology, Head and Neck Surgery, Chiba University Graduate School of Medicine, Chiba, Japan

**Keywords:** salivary duct carcinoma, survival, mGPS, CRP, NLR

## Abstract

The prognostic role of modified Glasgow Prognostic Score (mGPS), neutrophil-to-lymphocyte ratio (NLR) and platelet-to-lymphocyte ratio (PLR) in patients with salivary duct carcinoma (SDC) remains unclear. We conducted a multi-institutional retrospective cohort study of 140 SDC patients. The survival impact of these hematological markers was evaluated using multivariate proportional hazard models.High mGPS (≥1) was significantly associated with worse survival (3-year overall survival (OS): 16.7% vs 66.1%, *p*-value=0.003; 3-year progression-free survival (PFS): 0.0% vs 27.9%, *p*-value<0.001). Additionally, high C-reactive protein (CRP) (≥0.39 mg/dl) was significantly associated with worse survival (3-year OS: 32.1% vs 68.2%, *p*-value=0.001; 3-year PFS: 7.1% vs 31.1%, *p*-value<0.001). These associations were consistent with multivariate analysis adjusted for established prognostic factors. Although we also found significant association of high NLR (≥2.5) with OS (HR 1.80; 95% confidence interval, 1.05-3.08) in multivariate analysis, this association were inconsistent with the results of PFS. In addition, we found no significant associations of PLR with survival. In conclusion, we found that mGPS, CRP and NLR were identified as prognostic factors associated with survival in SDC patients.

## INTRODUCTION

Salivary duct carcinoma (SDC) arises from the ductal epithelium of the salivary gland and accounts for approximately 10% of all salivary gland malignancies [1, 2]. On pathological examination, the tumor resembles breast ductal carcinoma, and is characterized by ductal formation with a solid, cystic, cribriform, or papillary structure; elements of intraductal comedonecrosis; calcification; and a reactive desmoplastic stroma [1–4]. SDC is one of the most aggressive salivary gland malignancies, and prognosis remains poor due to the high incidence of locoregional recurrence and distant metastasis [5–11]. Several evaluations of clinical factors associated with survival in patients with SDC [4–10, 12–16] have reported prognostic values for age (<50 years) [12], primary tumor size [6, 7, 12, 15, 16], and lymph node involvement [6, 8, 12, 13, 15]. However, further detailed study of these prognostic factors is required to establish individual treatment strategies in SDC patients.

Recent studies have described the impact of several hematological inflammatory and nutritional markers on survival in a number of cancers, including head and neck cancer (HNC). These markers include the Glasgow prognostic score (GPS) or modified Glasgow prognostic score (mGPS), neutrophil-to-lymphocyte ratio (NLR) and platelet-to-lymphocyte ratio (PLR) [17–44]. These factors indicate nutritional and functional decline in patients with malignancies and are associated with poorer outcomes independent of clinical disease stage [20]. To date, however, the association between these factors and SDC survival has not been reported.

Here, to clearly identify these associations with adequate statistical power, we conducted a large-scale retrospective cohort study in a multi-institutional investigation setting in Japan.

## RESULTS

### Patient characteristics and survival

Table 1 summarizes the characteristics of the 140 SDC patients evaluated in this study. Median age was 64 years (range, 26–84 years) and median follow-up time was 3.3 years (range, 0.04–19.0 years). Males were predominant (86%). Primary tumor site was the parotid gland in 109 cases (78%), submandibular gland in 28 cases (20%), and others in 3 cases (2%). Regarding clinical disease stage, T4 and N2 were most common. Definitive surgery was performed for almost all cases. Carcinoma ex pleomorphic adenoma (CXPA) status was *de novo* in 53 cases (38%), invasive CXPA in 68 cases (49%), and non or micro invasive CXPA in 17 cases (12%). The 3-year overall survival (OS) among all patients was 65.5% (95% confidence interval (CI), 56.6–72.9), and 3-year progression-free survival (PFS) was 32.0% (95% CI, 24.3-40.0).

**Table 1:** Patient characteristics

Characteristics	N (=140)	%
**Age**		
Median (range)	64 (26-84)	
**Sex**		
Male	120	86
Female	20	14
**T classification**		
1	12	9
2	37	26
3	28	20
4a	61	44
4b	2	1
**N classification**		
0	65	46
1	8	6
2	66	47
3	1	1
**M classification**		
0	130	93
1	10	7
**Primary tumor site**		
Parotid gland	109	78
Submandibular gland	28	20
Others	3	2
**First-line treatment**		
Surgery	137	98
Radiotherapy	3	2
**CXPA status**		
*de novo*	53	38
CXPA invasion	68	49
CXPA non or micro invasion	17	12
unknown	2	1

Abbreviation: CXPA, carcinoma ex pleomorphic adenoma.

### Cut-off value of hematological markers in SDC patients

The cut-off value of hematological markers against clinical outcome in SDC patients was evaluated from the sensitivity, specificity, positive likelihood ratio, and negative likelihood ratio of the serial cut-off values. As only one patient had low albumin (<3.5 g/dl) in this study, we decided not to evaluate the optimal cut-off value of albumin. Optimal cut-off values of hematological markers were 0.39 mg/dl for C-reactive protein (CRP), 2.5 for NLR and 186.2 for PLR. Detailed information on all cut-off values are shown in Supplementary Table S1.

### Impact of hematological markers on survival

**Figure 1:**
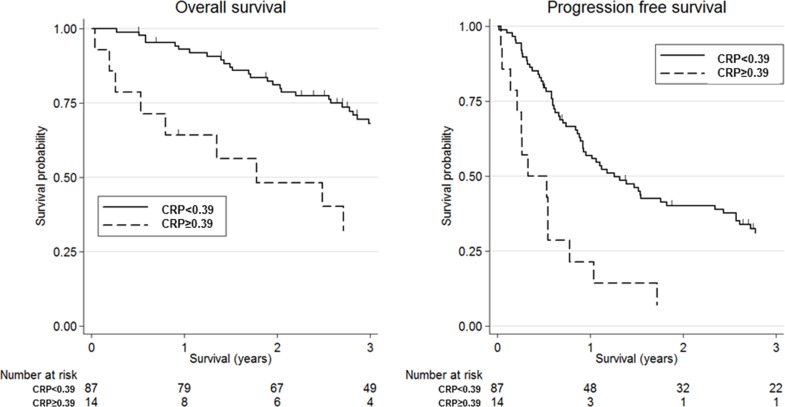
Kaplan-Meier survival curves according to modified Glasgow Prognostic Score (mGPS) Three-year overall survival was 16.7% (95% confidence interval (CI): 0.01-51.7) for high mGPS (≥1) and 66.1% (95% CI: 54.5-75.4) for low mGPS(=0) (log-rank test, *p*-value=0.003). Three-year progression-free survival was 0.0% for high mGPS and 27.9% (95% CI: 18.6-38.0) for low mGPS (log-rank test, *p*-value<0.001).

**Figure 2:**
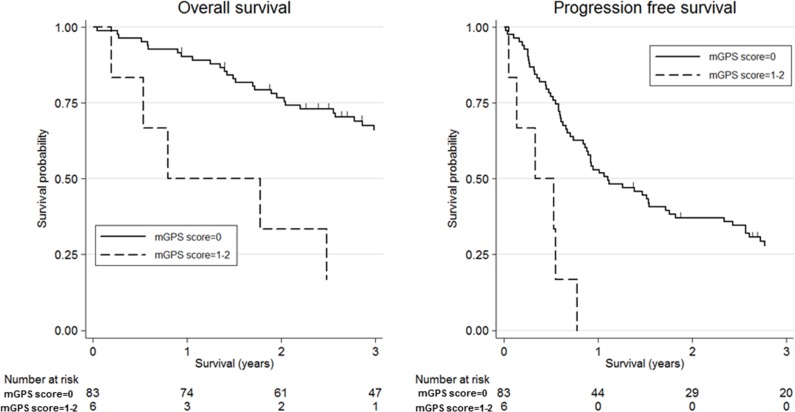
Kaplan-Meier survival curves according to C-reactive protein (CRP) Three-year overall survival was 32.1% (95% confidence interval (CI): 10.2-56.9) for high CRP (≥0.39 mg/dl) and 68.2% (95% CI: 56.8-77.1) for low CRP(<0.39 mg/dl) (log-rank test, *p*-value=0.001). Three-year progression-free survival was 7.1% (95% CI: 0.01-27.5) for high CRP and 31.1% (95% CI: 21.4-41.2) for low CRP (log-rank test, *p*-value<0.001).

Figure 1 shows the association between mGPS and clinical outcomes in patients with SDC using Kaplan-Meier survival curves. High mGPS (≥1) was significantly associated with worse OS and PFS compared with low mGPS (=0) [3-year OS: 16.7% (95% CI, 0.01-51.7) vs 66.1% (95% CI, 54.5-75.4), *p*-value=0.003; 3-year PFS: 0.0% vs 27.9% (95% CI, 18.6-38.0), *p*-value<0.001]. Additionally, Figure 2 shows the association between CRP and clinical outcomes in patients with SDC using Kaplan-Meier survival curves; as with mGPS, high CRP (≥0.39 mg/dl) was significantly associated with worse OS and PFS compared with low CRP (<0.39 mg/dl) [3-year OS: 32.1% (95% CI, 10.2-56.9) vs 68.2% (95% CI, 56.8-77.1), *p*-value=0.001; 3-year PFS: 7.1% (95% CI, 0.01-27.5) vs 31.1% (95% CI, 21.4-41.2), *p*-value<0.001].

Table 2 shows the results of uni- and multivariate analysis of hematological markers for clinical outcome in SDC patients. In multivariate analysis adjusted by other general clinical factors, hazard ratios (HRs) for the high mGPS (≥1) group relative to the low group (=0) were 4.68 (95% CI, 1.22–17.91; *p*-value=0.024) for OS and 3.92 (95% CI, 1.23–12.49; *p*-value=0.021) for PFS, respectively. In addition, HRs for the high CRP group (≥0.39 mg/dl) relative to the low group (<0.39 mg/dl) were 2.45 (95% CI, 1.14–5.30; *p*-value=0.022) for OS and 2.53 (95% CI, 1.28–5.00; *p*-value=0.007) for PFS, respectively. Although we found significant association of high NLR (≥2.5) with OS (HR 1.80; 95% CI, 1.05-3.08; *p*-value=0.032), this association was inconsistent with the results of PFS. Regarding to PLR, we found no significant associations with survival.

**Table 2:** Univariate and multivariate analysis of clinical outcomes in patients with salivary duct carcinoma

Inflammatory markers	N	Overall survival						Progression-free survival					
		Univariate analysis			Multivariate analysis			Univariate analysis			Multivariate analysis		
		HR	95% CI	*p*-values	HR	95% CI	*p*-values	HR	95% CI	*p-*values	HR	95% CI	*p*-values
**mGPS**													
0	83	1.00	-	reference	1.00	-	reference	1.00	-	reference	1.00	-	reference
1-2	6	3.83	1.50-9.80	0.005	4.68	1.22-17.91	0.024	4.66	1.95-11.13	0.001	3.92	1.23-12.49	0.021
unknown	51	0.92	0.55-1.54	0.745	1.32	0.75-2.34	0.340	0.73	0.47-1.13	0.155	0.85	0.52-1.38	0.509
**CRP (mg/dl)**													
<0.39	87	1.00	-	reference	1.00	-	reference	1.00	-	reference	1.00	-	reference
≥0.39	14	3.04	1.55-5.97	0.001	2.45	1.14-5.30	0.022	2.93	1.59-5.38	0.001	2.53	1.28-5.00	0.007
unknown	39	1.08	0.62-1.88	0.791	1.86	0.99-3.51	0.056	0.80	0.50-1.28	0.350	1.03	0.62-1.72	0.896
**NLR**													
<2.5	84	1.00	-	reference	1.00	-	reference	1.00	-	reference	1.00	-	reference
≥2.5	49	1.98	1.20-3.25	0.007	1.80	1.05-3.08	0.032	1.27	0.84-1.93	0.261	1.00	0.63-1.59	0.994
unknown	7	2.40	0.99-5.81	0.053	2.57	0.95-6.95	0.063	1.39	0.60-3.24	0.442	1.50	0.61-3.71	0.378
**PLR**													
<186.2	105	1.00	-	reference	1.00	-	reference	1.00	-	reference	1.00	-	reference
≥186.2	28	1.98	1.15-3.39	0.013	1.82	0.98-3.36	0.057	1.36	0.83-2.22	0.219	1.04	0.61-1.78	0.885
unknown	7	2.17	0.91-5.15	0.080	2.47	0.92-6.62	0.072	1.35	0.59-3.12	0.477	1.52	0.62-3.74	0.361

Adjusted by age, sex, primary tumor site, TNM classification, first-line treatment, CXPA status.

Abbreviations: mGPS, modified Glasgow Prognostic Score; CRP, C-reactive protein; NLR, neutrophil-to-lymphocyte ratio; PLR, platelet-to-lymphocyte ratio; HR, hazard ratio; CXPA, carcinoma ex pleomorphic adenoma; CI, confidence interval.

### Interaction between mGPS, CRP and other clinical factors

We examined interactions between mGPS, CRP and other clinical factors of SDC (Table 3). For OS, no significant interactions with other clinical factors were found. However, for PFS, significant interactions were observed between mGPS, primary tumor site and N classification, and CRP and sex. For mGPS, the impact of high mGPS on PFS was stronger in patients with the parotid gland and N0. In addition, because the number of cases was low, we could not estimate the ORs of high CRP for females. Finally, HRs for both mGPS and CRP were higher in CXPA cases than in *de novo* cases, albeit without significance.

**Table 3:** Interaction between systemic inflammatory markers and clinical characteristics on clinical outcomes in patients with salivary duct carcinoma

Characteristics	Overall survival										Progression-free survival									
	mGPS (≥1)					CRP (≥0.39 mg/dl)					mGPS (≥1)					CRP (≥0.39 mg/dl)				
	N	HR	95% CI	*p*-values	*p*_for heterogeneity_	N	HR	95% CI	*p*-values	*p*_for heterogeneity_	N	HR	95% CI	*p*-values	*p*_for heterogeneity_	N	HR	95% CI	*p*-values	*p*_for heterogeneity_
**Age**																				
<65	45	6.94	0.61-79.36	0.119	0.623	52	2.55	0.63-10.34	0.189	0.623	45	5.20	0.89-30.42	0.068	0.869	52	1.64	0.53-5.13	0.394	0.364
≥65	44	3.36	0.34-33.08	0.299		49	3.00	1.00-9.04	0.051		44	4.44	0.39-50.66	0.230		49	3.83	1.46-10.07	0.006	
**Sex**																				
Male	78	4.03	1.10-14.80	0.036	0.544	87	2.41	1.06-5.47	0.036	0.106	78	4.14	1.39-12.38	0.011	0.441	87	1.94	0.97-3.89	0.062	0.034
Female	11	NE	-	-		14	NE	-	-		11	NE	-	-		14	NE	-	-	
**Primary tumor site**																				
Parotid gland	65	20.23	3.27-125.17	0.001	0.103	75	3.78	1.58-9.03	0.003	0.144	65	28.26	5.27-151.64	<0.001	0.045	75	2.87	1.28-6.48	0.011	0.344
Others	24	2.52	0.09-70.79	0.586		26	1.13	0.10-13.19	0.922		24	3.43	0.46-25.85	0.231		26	1.35	0.32-5.68	0.682	
**T classification**																				
1-2	32	21.82	2.01-236.49	0.011	0.377	35	6.65	0.85-52.12	0.072	0.531	32	22.79	2.24-231.49	0.008	0.817	35	12.52	1.47-106.67	0.021	0.662
3-4	57	4.64	0.65-33.01	0.125		66	2.85	1.23-6.57	0.014		57	3.24	0.81-12.98	0.097		66	2.71	1.32-5.56	0.006	
**N classification**																				
0	40	51.96	5.04-535.29	0.001	0.103	43	5.41	1.04-28.24	0.045	0.377	40	220.04	17.81-2718.82	<0.001	0.045	43	10.72	2.12-54.04	0.004	0.128
>1	49	1.92	0.18-20.62	0.590		58	2.49	0.95-6.51	0.064		49	1.84	0.33-10.18	0.486		58	1.65	0.71-3.82	0.247	
**M classification**																				
0	81	3.20	0.68-15.03	0.141	0.316	93	2.44	1.10-5.41	0.028	0.400	81	4.35	1.13-16.70	0.032	0.632	93	2.09	1.01-4.36	0.048	0.653
1	8	NE	-	-		8	NE	-	-		8	NE	-	-		8	NE	-	-	
**First-line treatment**																				
Surgery	86	4.33	1.18-15.91	0.027	0.886	98	2.85	1.33-6.11	0.007	0.551	86	3.53	1.09-11.38	0.035	0.999	98	2.22	1.13-4.34	0.020	0.734
Radiotherapy	3	NE	-	-		3	NE	-	-		3	NE	-	-		3	NE	-	-	
**CXPA status**																				
*de novo*	35	1.34	0.08-23.80	0.841	0.501	38	1.81	0.49-6.76	0.375	0.456	35	1.07	0.13-8.43	0.952	0.588	38	1.31	0.41-4.19	0.649	0.425
CXPA	53	7.99	1.34-47.72	0.023		62	4.98	1.72-14.44	0.003		53	3.51	0.61-20.32	0.161		62	3.02	1.12-8.14	0.029	

Abbreviations: mGPS, modified Glasgow Prognostic Score; CRP, C-reactive protein; CI, confidence interval; CXPA, carcinoma ex pleomorphic adenoma; NE, not estimatable.

## DISCUSSION

In this study, we found that high mGPS, CRP and NLR before treatment were significantly associated with poor survival in SDC patients. To our knowledge, this is the first study to identify these associations and suggests that the investigation of mGPS, CRP and NLR before treatment may be effective for the selection of high-risk SDC patients.

Although the mechanism behind these associations between hematological markers associated with systemic inflammation and nutrition and the prognosis of SDC is unclear, some possibilities can be mentioned based on previous evidence. First, elevated these hematological markers may be surrogate markers of pre-cancer cachexia, which is characterized by increased weight loss, poor performance status, increased comorbidity, increased pro-inflammatory and angiogenic cytokines, and complications on treatment [20]. It has been noted that cancer cachexia is associated with poor survival [45–47]. Second, inflammation in the tumor microenvironment causes a range of aggressive tumor behaviors, including proliferation and survival of tumor cells, promotion of metastasis and angiogenesis, host immune deficiency, and alteration of responses to hormones and chemotherapeutic agents [48, 49].

To date, we are aware of at least 23 studies that have evaluated the association between GPS or mGPS, NLR, PLR and survival in patients with HNC [22–44]. Although most studies indicated that these factors had significant prognostic impact, one study each of NLR [25] and PLR [44] showed no significant association with survival. In the present study, although we detected significant association of NLR with OS in SDC patients, we could not find significant association with PFS. According to PLR, We also found the similar association with OS and PFS. Therefore, we evaluated the associations of NLR and PLR with disease-specific survival (DSS), and found significant associations of these factors with DSS (data not shown). According to this results, we interpreted that NLR and PLR might be associated with poor survival after progressin of tumor. Although the reason for this inconsistency is difficult to determine, a recent study suggested that mGPS might be superior to NLR and PLR in patients with nasopharyngeal cancer [22]. Additionally, since our cohort included only one case with low albumin, CRP might be a better indicator of prognosis than mGPS in patients with SDC. These findings indicate that systemic inflammatory and nutritional conditions should be evaluated before treatment. This information might be valuable in the development of individual treatment strategies for patients with SDC, including appropriate supportive care.

Our study has two methodological strengths. First, the clinicians involved in the care of study patients had no information on the association between hematological markers and SDC survival, which likely precluded the introduction of information bias. Second, our sample size is one of the largest among cohort studies in patients with SDC.

Additionally, several limitations of this study warrant mention. First, our information on hematological markers reflected pretreatment status only. Second, we could not completely remove the possibility of infection or other inflammatory conditions. Third, although our sample size represents one of the largest cohorts in SDC, the relatively large amount of missing data may have limited the statistical power.

In conclusion, we found significant positive associations of mGPS, CRP and NLR with survival in patients with SDC. These findings provide evidence in support of the development of individual treatment strategies in SDC.

## MATERIALS AND METHODS

### Patients

The study was conducted under a retrospective cohort design in patients with SDC treated at seven hospitals between 1992 and 2014: the International University of Health and Welfare Mita Hospital, Keio University Hospital, Hokkaido University Hospital, Tokyo Medical University Hospital, Tokyo Medical University Hachioji Medical Center, Tokai University Hospital, and Niigata Cancer Center Hospital. The study design was approved by the Institutional Ethics Review Board of each of these hospitals. One hundred and forty SDC patients were enrolled in the study.

### Treatment and follow-up

All samples underwent central pathological review by two expert pathologists (T.N. and Y.S.). Diagnoses were conducted independently, and any disagreements were resolved by discussion. Staging was in accordance with the UICC TNM classification and staging system (2010, 7th edition). Given that no effective chemotherapy regimen for patients with SDC has yet been established, operable cases were treated by definitive surgery with or without neck dissection [6]. Postoperative radiotherapy was performed when the surgical margin was positive or equivocal and/or lymph node metastasis was pathologically positive. For inoperable cases, we performed palliative radiotherapy or chemotherapy, in consideration of performance status (PS) and comorbidities. Following the end of treatment, patients underwent a medical history and physical examination, complete blood cell count, and imaging examination every 3–6 months. Vital and disease status were confirmed by checking medical records at the date of the last follow-up visit.

### Evaluation of hematological inflammatory and nutritional markers

In this study, we selected several pre-treatment hematological markers associated with systemic inflammatory and nutritional condition, including CRP, serum albumin, complete blood count with circulating neutrophil count, circulating lymphocyte count, circulating platelet count, NLR, and PLR. Regarding mGPS, patients with both an elevated CRP level (>1.0 mg/dl) and lower albumin (<3.5 g/dl) were allocated a score of 2; those with an elevated CRP level (>1.0 mg/dl) and non-decreased albumin (≥3.5 g/dl) were allocated a score of 1; and those with a non-elevated CRP level (≤1.0 mg/dl) were allocated a score of 0 [20].

### Statistical analysis

Cut-off values for continuous variables of hematological markers in SDC patients were evaluated using the area under the receiver operating characteristic curve (AUROC). We defined overall death as the objective standard and continuous values of the hematological markers as the diagnostic test value.

Primary endpoint was OS, defined as the interval between the beginning of treatment and the date of death or last follow-up. PFS was measured as a secondary endpoint and defined as the number of days from the beginning of treatment to the date of relapse or progression, as evaluated and recorded by the attending physician. The association between hematological markers, OS, and PFS was evaluated by the Kaplan–Meier product-limit method and univariate and multivariate Cox proportional hazards models. The measure of association in this study was HR with a 95% CI. Confounders considered in the univariate and multivariate analyses were age (<65 vs ≥65), sex (male vs female), T classification (1/2/3/4), N classification (0/1/2/3), M classification (0/1), primary tumor site (parotid gland vs submandibular gland vs others), first-line treatment (surgery vs radiotherapy), and CXPA status (de novo vs invasive CXPA vs non or micro invasive CXPA). All statistical analyses were performed using STATA version 13 (Stata Corp., College Station, TX, USA). All tests were two-sided, and p values of <0.05 were considered statistically significant.

## SUPPLEMENTARY TABLE


