# Supplementary material for: Impact of hematological inflammatory markers on clinical outcome in patients with salivary duct carcinoma: a multi-institutional study in Japan
Source: Oncotarget. 2016 Nov 24;8(1):1083–91. doi: 10.18632/oncotarget.13565 (PMC5352036; doi:10.18632/oncotarget.13565)
Supplement: Supplementary file 1 [file oncotarget-08-1083-s001.pdf]

Impact of hematological inflammatory markers on clinical outcome in patients with salivary duct carcinoma: a multi-institutional study in Japan

SUPPLEMENTARY TABLE

Supplementary Table S1: Impact of diagnostic value of hematological markers in patients with salivary duct carcinoma

Optimal cut-off value	Sensitivity	Specificity	LR^+	LR^-
**CRP**				
0.39 mg/dl	25.0	94.3	4.42	0.80
**NLR**				
2.5	49.2	74.3	1.91	0.68
**PLR**				
186.2	31.8	87.1	2.47	0.78

Abbreviations: CRP, C-reactive protein; NLR, neutrophil-to-lymphocyte ratio; PLR, platelet-to-lymphocyte ratio; LR, likelihood ratio.
